# Reduction of carcinogens in fermented fish (pla-ra and pla-som) by heating

**DOI:** 10.14202/vetworld.2023.1727-1735

**Published:** 2023-08-25

**Authors:** Atchara Artchayasawat, Pranee Sriraj, Thidarut Boonmars, Ratchadawan Aukkanimart, Ampas Wisetmora, Glenn N. Borlace, Parichart Boueroy, Benjamabhorn Pumhirunroj, Porntip Laummaunwai, Panaratana Rattanasuwan, Sirintip Boonjaraspinyo, Nattapon Ekobol, Opal Pitaksakulrat, Wu Zhiliang

**Affiliations:** 1 Department of Parasitology, Faculty of Medicine, Khon Kaen University, Khon Kaen, 40002, Thailand; 2 Cholangiocarcinoma Research Institute, Khon Kaen University, Khon Kaen, 40002, Thailand; 3 Department of Traditional Medicine, Faculty of Natural Resources, Rajamangala University of Technology ISAN Sakon Nakhon Campus, Sakon Nakhon, 47160, Thailand; 4 Department of Pharmaceutical Chemistry, Faculty of Pharmaceutical Sciences, Khon Kaen University, Khon Kaen, 40002, Thailand; 5 Department of Community Health, Faculty of Public Health, Kasetsart University Chalermphakiat Sakon Nakhon Province Campus, Sakon Nakhon, 47000, Thailand; 6 Program in Animal Science, Faculty of Agricultural Technology, Sakon Nakhon Rajabhat University, Sakon Nakhon, 47000, Thailand; 7 Department of Anesthesiology, Faculty of Medicine, Khon Kaen University, Khon Kaen, 40002, Thailand; 8 Department of Community Medicine, Family Medicine and Occupational Medicine, Faculty of Medicine, Khon Kaen University, Khon Kaen, 40002, Thailand; 9 Department of Parasitology and Infectious Disease, Graduate School of Medicine, Gifu University, Japan

**Keywords:** cholangiocarcinoma, fish, liver flukes, opisthorchiasis

## Abstract

**Background and Aim:**

The risk factors for cholangiocarcinoma (CCA) are opisthorchiasis and the intake of a combination of nitroso compounds through the consumption of traditionally fermented fish, which is very popular in areas where liver flukes are endemic. The incidence of CCA remains high because this cultural habit of rural people has been altered. Therefore, decreasing nitrate and nitrite concentrations in fermented fish are an alternative approach to reducing the risk of CCA. Thus, this study aimed to reduce nitrate and nitrite concentrations in fermented foods by heating and investigated its effect on CCA development in a hamster model.

**Materials and Methods:**

We used Association of Official Analytical Chemists method 973.31 to measure the nitrate and nitrite concentrations in both fermented fish (pla-ra [PR]) and pickled fish (pla-som [PS]) before and after boiling for 5 and 30 min, respectively. The same samples were fed to *Opisthorchis* *viverrini* (OV)-infected or -uninfected hamsters for 3 months. Thereafter, the hamsters’ liver and blood were collected for analysis.

**Results:**

The levels of nitrates and nitrites in PS and PR significantly decreased following boiling for 5 and 30 min. The OV-PR and OV-PS groups showed dramatically increased numbers of inflammatory cells, fibrosis surrounding the bile duct, and focal fibrotic areas. However, after boiling the fermented dishes for 5 and 30 min, the extent of inflammatory cell infiltration and intensity of fibrosis in these groups were decreased.

**Conclusion:**

Our findings suggest that boiling reduces nitrate and nitrite toxicity in fermented dishes, as evidenced by reduced hepatic inflammation. However, regardless of heating, kidney tissues are adversely affected when fermented meals are consumed daily.

## Introduction

Cholangiocarcinoma (CCA) is one of the most common cancers in the Lao People’s Democratic Republic (Lao PDR), Cambodia, and Thailand, particularly in the northeastern region, where the prevalence of liver fluke infection is high [1–3]. In these countries, the major causes of CCA are the contamination of fermented foods by abiotic factors (nitroso compounds, i.e., nitrates, nitrites, and nitrosamines) and biotic factors (i.e., *Opisthorchis viverrini* [OV] infections in raw fish) [4]. In northeast Thailand, pla-ra (PR) and pla-som (PS) are the popular traditional dishes that contain fermented fish. Nitrates and nitrites are specifically used in food preparation to inhibit *Clostridium botulinum* growth [5–7] and lipid peroxidation [8]. However, the use of nitrites as curing agents for meat is a public concern because they can be a precursor of nitrosamines, many of which are known carcinogens [9]. In 2010, the International Agency for Research on Cancer reported that even the daily intake of low levels of nitrate (<50 mg/L) could lead to colorectal cancer in humans [4]. Furthermore, a nitrite concentration of 1000 ppm has been reported to cause hepatocellular carcinoma in a hamster model [10]. *N*-nitrosamine forms during the meat preservation process, which uses nitrite and nitrate. This major group of nitrosamines leads to many cancers, such as bladder cancer [11], colorectal cancer [12–14], stomach cancer [15], and pancreatic cancer [16], as well as CCA [17].

Chinese-style salted fish is classified as a Group 1 carcinogen due to the formation of *N*-nitroso compounds during the preparation and steaming processes and causes nasopharyngeal cancer in humans [18]. Hamsters treated with *N*-nitrosodimethylamine (NDMA) and OV have been used as animal models for CCA studies in Thailand [19, 20]. Gas chromatography has revealed high NDMA levels in fermented fish dishes, such as PR and PS, and in mixed fermented vegetables and fermented beef [21]. *Opisthorchis viverrini* infection and fermented foods have been linked to CCA development [22].

Several approaches to decreasing nitrate, nitrite, and nitrosamine concentrations in foods have been proposed, such as antioxidant treatment, pH reduction, and heating [23, 24]. Therefore, this study aimed to determine the effect of heating on the reduction of nitrate and nitrite levels in fermented foods and its effect on CCA development in a hamster model.

## Materials and Methods

### Ethical approval

All protocols, including housing and animal laboratory management, were approved by the Animal Ethics Committee, Khon Kaen University (AEKKU 47/2556 and AEMDKKU 007/2022).

### Study period and location

The study was conducted from May 2016 to December 2022. This study was conducted in the Department of Parasitology, Faculty of Medicine, Khon Kaen University, Thailand.

### Animals

Seventy male Syrian hamsters (n = 5 per group), aged 6–8 weeks were obtained from the Animal Unit, Faculty of Medicine, Khon Kaen University, and randomly selected for use in each experimental group.

### Experimental design

This experiment studied the effect of different boiling times on nitrate and nitrite levels reduction in fermented foods that were subsequently fed to Syrian hamsters infected with OV metacercariae for 3 months post-infection. There were six major experimental groups: (i) Normal (uninfected) hamsters (N), (ii) normal hamsters fed the fermented fish dish PR (N-PR), (iii) normal hamsters fed the pickled fish dish PS (N-PS), (iv) hamsters infected with OV, (v) hamsters infected with OV and fed PR (OV-PR), and (vi) hamsters infected with OV and fed PS (OV-PS).

There were 14 subgroups: (i) Normal (uninfected) hamsters (N), (ii) normal hamsters fed PR (N-PR), (iii) normal hamsters fed PR that had been boiled for 5 min (N-PR5), (iv) normal hamsters fed PR that had been boiled for 30 min (N-PR30), (v) normal hamsters fed PS (N-PS), (vi) normal hamsters fed PS that had been boiled for 5 min (N-PS5), (vii) normal hamsters fed PS that had been boiled for 30 min (N-PS30), (viii) OV-infected hamsters (OV), (ix) OV-infected hamsters fed PR (OV-PR), (x) OV-infected hamsters fed PR that had been boiled for 5 min (OV-PR5), (xi) OV-infected hamsters fed PR that had been boiled for 30 min (OV-PR30), (xii) OV-infected hamsters fed PS (OV-PS), (xiii) OV-infected hamsters fed PS that had been boiled for 5 min (OV-PS5), and (xiv) OV-infected hamsters fed PS that had been boiled for 30 min (OV-PS30).

The hamsters’ liver and blood were collected after 3 months of treatment and used for histopathological analysis and liver and kidney function assessment, respectively.

### Preparation of PR and PS

PR and PS were purchased from a local market in Khon Kaen province, Thailand (16° 26’ 17.8” N, 102° 47’ 59.5” E). PR is a raw fermented fish dish that has been fermented for at least 6 months and mainly comprises fish and salt. Pla-som is a raw pickled fish dish that has been fermented for at least 3 days and mainly comprises fish, garlic, salt, and rice. Both PR and PS were blended with distilled water at a ratio of 1:4 and then boiled at 80°C for either 5 or 30 min. The pH was determined before and after boiling. The fish solutions were stored in aliquots of 15 mL at −20°C. Before use, each sample was thawed at 25°C and then fed to the assigned groups daily for 3 months (0.5 mL/hamster/d).

### Determination of nitrite and nitrate levels

The nitrite and nitrate concentrations in the fish solutions were analyzed by Central Laboratory (Thailand) Co., Ltd., Khon Kaen, using an in-house method based on Association of Official Analytical Chemists 973.31. Briefly, 5 g of minced sample was mixed with hot water, transferred into a volumetric flask, boiled in a water bath (80°C) for 2 h, and allowed to cool to 25°C. The volume was made up, and the sample was centrifuged. In a 50 mL volumetric flask, 2.5 mL of sulfanilamide reagent and 2.5 mL of N-(1-naphthyl) ethylenediamine (NED) reagent were added. Then, the color was allowed to develop for 15 min. Finally, an ultraviolet-visible spectrophotometer measured the sample absorbance at 540 nm against the reagent blank. The amount of nitrite present was determined using a standard curve. A set of working standard solutions containing 10, 20, 30, and 40 mL of the nitrite standard solution was prepared. Then, sulfanilamide and NED were added to the working standard solutions using the same method as for the samples, and the absorbance was read at 540 nm. The results were plotted against the corresponding concentrations of the nitrite standard solutions [25].

### *Opisthorchis viverrini* metacercariae preparation

*Opisthorchis viverrini* metacercariae were obtained from naturally infected cyprinoid fish that were collected from endemic areas in the Lao PDR. The fish were minced in an electric blender and mixed with 0.25% pepsin A solution at a ratio of 1:3 (v/v). The mixture was incubated at 37°C in a shaking water bath for 1 h and filtered through a descending series of four sieves with pore sizes of 1000, 300, 250, and 106 μm, respectively. Finally, the filtrates were placed in a sedimentary jar with 0.85% NaCl until the supernatant was clear. Then, the sediments were examined under a dissecting microscope (Nikon, Japan) for the presence of mature OV metacercariae. Groups of 50 active metacercariae were used to infect the hamsters by intragastric intubation on day 0.

### Animal sacrifice and specimen collection

Syrian hamsters from each group were sacrificed after 3 months of treatment. Whole blood was collected by cardiac puncture and drawn into a 1.5 mL tube. Following centrifugation at 9,100 × *g* at 4°C for 10 min to separate the serum from packed red cells, the serum was separated and stored at –20°C until use in the liver and kidney function tests. The liver was removed and fixed in 10% formalin for histopathological study.

### Histopathological analysis of liver tissue

After dissection and fixation in 10% formalin, the liver tissues were washed with phosphate-buffered saline for 12 h and prepared for histopathological study using a conventional protocol. First, they underwent dehydration in an ascending series of alcohol as follows: 70% and 80% alcohol for 1 h (two times), 95% alcohol for 1 h (three times), and absolute alcohol for 1 h (3 times). The tissues were cleared in xylene for 1 h (two times) and paraffinized in an incubator at 60°C with paraffin solution for 1.5 and 2 h, respectively. Then, the embedded tissues were cut into 4 μm-thin sections using a microtome and placed on microscope slides. Finally, the tissue section slides were incubated at 60°C for 24 h and stored at room temperature (30°C).

Before staining with H&E, the tissue sections were deparaffinized in xylene, rehydrated through a descending alcohol series, washed with distilled water, and stained with Harris hematoxylin, followed by eosin. Then, the sections were dehydrated through an ascending series of ethanol, cleared in xylene, and mounted on slides using a Permount mounting medium (Fisher Chemical, UK). The liver sections were examined and digitized using an Olympus BX51 light microscope (Tokyo, Japan) for pathological grading.

For scoring, the histopathological study focused on periportal hepatic tissue inflammation and focal areas of hepatic inflammation. Scoring of periportal or periseptal interface hepatitis (piecemeal necrosis) was as follows: 0: None, 1: Mild (focal, few portal areas), 2: Mild/moderate (focal, most portal areas), 3: Moderate (continuous around <50% of tracts or septa), and 4: Severe (continuous around >50% of tracts or septa). Focal inflammation was scored as follows: 0: None, 1: ≤1 foci/×10 objective, 2: 2–4 foci/×10 objective, 3: 4–10 foci/×10 objective, and 4: ≥10 foci/×10 objective [26].

To assess fibrogenesis in the liver tissue, sections were dyed with 0.1% Sirius red (Sigma-Aldrich, St. Louis, MO, USA) for 1 h, washed with 1% (v/v) acetic acid, and serially dehydrated in ethanol as before. Then, the liver sections were photographed at 10× magnification using an Olympus BX51 light microscope, and the degree of fibrosis was graded using the following scoring criteria [22–24]: 0: No fibrosis, 1: Fibrous expansion of some portal areas ± short fibrous septa, 2: Fibrous expansion of most portal areas ± short fibrous septa, 3: Fibrous expansion of most portal areas ± occasional portal to portal (P–P) bridge, 4: Fibrous expansion of the portal area ± marked P–P and portal to central (P–C) bridging, 5: Marked P–P and P–C bridging with occasional nodules (incomplete), and 6, cirrhosis [26–28].

### Liver and kidney function tests

The serum alanine aminotransferase (ALT) and alkaline phosphatase (ALP) levels are sensitive markers of liver tissue injury and were determined. Blood urea nitrogen (BUN) and creatinine levels were determined to assess the effect of the fermented dishes on kidney function. The serum ALT, ALP, BUN, and creatinine levels were analyzed by the community laboratory, Faculty of Associated Medical Science, Khon Kaen University.

### Statistical analysis

The histopathological data were expressed as scores of 0–4. Liver, kidney, nitrate, and nitrite levels were expressed as the mean ± standard deviation. All data analyses were performed using the statistical package for the social sciences (SPSS) v16.0 statistical software (SPSS, Inc., Chicago, IL, USA). Statistically significant differences were considered when p < 0.05.

## Results

### Nitrate and nitrite levels

The nitrate levels in PR and PS were <20.00 mg/kg before boiling. In either PR or PS, nitrates were not detected in following boiling at 80°C for 5 and 30 min. Prior boiling, the nitrite levels in PR and PS were <5.00 mg/kg. After boiling at 80°C for 5 and 30 min, respectively, nitrites were not detected in PR or PS boiled for 30 min but were detected at levels <5.00 mg/in PS boiled for 5 min (Table-1). In the fermented fish and pickled fish groups that were boiled for boiling for 5 and 30 min groups, the pH was lower compared with the raw fermented food groups (p < 0.05; Table-1).

**Table-1:** The nitrate, nitrite, and pH level before and after boiling at different time points.

Groups	Nitrate (mg/kg)	Nitrite (mg/kg)	pH
PR	<20.00	<5.00	5.04 ± 0.02
PR 5 min	Not detected	Not detected	4.84 ± 0.02*
PR 30 min	Not detected	Not detected	4.60 ± 0.05*
PS	<20.00	<5.00	4.97 ± 0.03
PS 5 min	Not detected	<5.00	4.88 ± 0.01^#^
PS 30 min	Not detected	Not detected	4.34 ± 0.02^#^

PR=Raw fermented fish, PR 5 min=Fermented fish with boiling for 5 min, PR 30 min=Fermented fish with boiling for 30 min, PS=Raw pickled fish, PS 5 min=Pickled fish with boiling for 5 min, PS 30 min=Pickled fished with boiling for 30 min,

* p < 0.05 when compared with PR group,

# p < 0.05 when compared with PS group

### Histopathological changes

**Figure-1:**
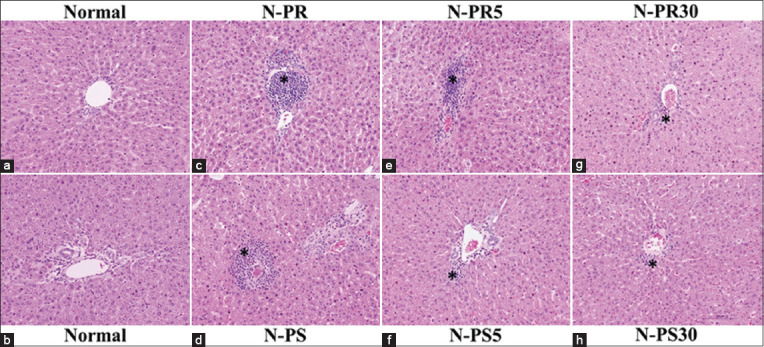
Histopathological changes in the hepatobiliary system of the experimental groups. (a and b) Normal: Untreated hamster, (c) N-PR=Normal hamsters feed the raw fermented fish, (d) N-PS=Normal hamsters feed the raw pickled fish, (e) N-PR5=Normal hamsters feed the fermented fish with boiling for 5 min, (f) N-PS5=Normal hamsters feed the pickled fish with boiling for 5 min, (g) N-PR30=Normal hamsters feed the fermented fish with boiling for 30 min, and (h) N-PS30=Normal hamsters feed the pickled fish with boiling for 30 min, *Inflammatory cells aggregation.

**Figure-2:**
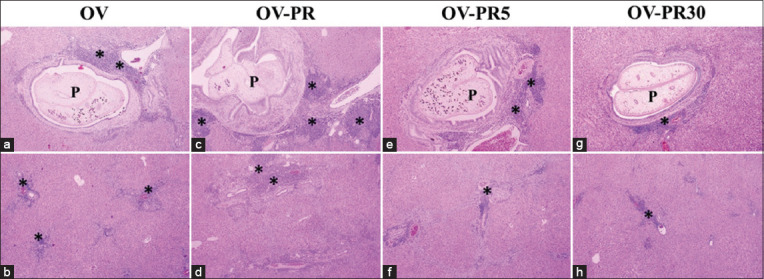
Histopathological changes in the hepatobiliary system of the experimental groups. (a and b) OV=Hamster infected with *Opisthorchis viverrini*, (c and d) OV-PR=Opisthorchiasis’s hamsters feed the fermented fish, (e and f) OV-PR5=opisthorchiasis’s hamsters feed the fermented fish with boiling for 5 min, (g and h) OV-PR30=Opisthorchiasis’s hamsters feed the fermented fish with boiling for 30 min, P=Parasite (*O. viverrini*) in bile duct, *inflammatory cells aggregation.

**Figure-3:**
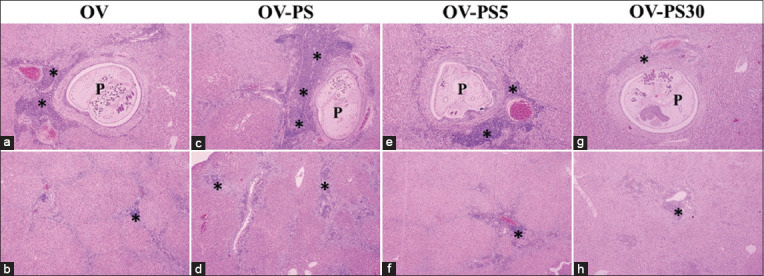
Histopathological changes in the hepatobiliary system of the experimental groups. (a and b) OV=Hamster infected with *Opisthorchis viverrini*, (c and d) OV-PS=Opisthorchiasis’s hamsters feed the pickled fish, (e and f) OV-PS5=Opisthorchiasis’s hamsters feed the boiled pickled fish for 5 min, and (g and h) OV-PS30=Opisthorchiasis’s hamsters feed the boiled pickled fish for 30 min, P=Parasite (*O. viverrini*) in bile duct, *inflammatory cells aggregation.

The histopathological changes caused by nitroso compounds (nitrates and nitrites) were examined in OV-infected hamsters. The analysis focused on the aggregation of inflammatory cells surrounding the intrahepatic bile ducts and focal inflammation. The normal group (Figures-1a and b) were histologically normal (no inflammatory cells surrounding the intrahepatic bile ducts). The N-PR and N-PS groups showed aggregations of inflammatory cells surrounding the intrahepatic bile ducts (Figures-1c and d). Only a few inflammatory cells surrounding the intrahepatic bile ducts were observed in all groups fed fermented fish that had been boiled (Figures-1e–h). The OV-PR and OV-PS groups showed the highest aggregation of inflammatory cells surrounding the intrahepatic bile ducts and more areas of focal inflammation compared with the OV, OV-PR5, OV-PR30, OV-PS5, and OV-PS30 groups. In groups fed PR and PS that had been boiled for 30 min, the aggregation of inflammation cells was nearly by OV group (Figures-2 and 3). Tables-2 and 3 show the results of the grading of histopathological changes (periportal inflammation, focal inflammation, and liver fibrosis) in each group of hamsters.

**Table-2:** Histopathological features of liver grading criteria in normal group infection.

Histopathology	Score	Groups of experiment						

		Normal	N-PR	N-PR5	N-PR30	N-PS	N-PS5	N-PS30
						
		N = 5	N = 5	N = 5	N = 5	N = 5	N = 5	N = 5
						
		% (N)	% (N)	% (N)	% (N)	% (N)	% (N)	% (N)
Periportal	0	100 (5)^a,b^		40 (2)	80 (4)		80 (4)	80 (4)
Inflammation	1		40 (2)	60 (3)^a^	20 (1)^a^	60 (3)	20 (1)^b^	20 (1)^b^
	2		60 (3)			40 (2)		
	3							
	4							
Focal	0	100 (5)^a,b^	40 (2)	100 (5)^a^	100 (5)^a^	40 (2)	100 (5)^b^	100 (5)^b^
Inflammation	1		60 (3)			60 (3)		
	2							
	3							
	4							
Liver fibrosis	0	100 (5)	100 (5)	100 (5)	100 (5)	100 (5)	100 (5)	100 (5)
	1							
	2							
	3							
	4							
	5							
	6							

N=Number of hamster,

a p < 0.05 compared with N-PR group,

b p < 0.05 compared with N-PS group. N-PR=Normal hamsters feed the raw fermented fish, N-PS=Normal hamsters feed the raw pickled fish, N-PR5=Normal hamsters feed the fermented fish with boiling for 5 min, N-PS5=Normal hamsters feed the pickled fish with boiling for 5 min, N-PR30=Normal hamsters feed the fermented fish with boiling for 30 min, N-PS30=Normal hamsters feed the pickled fish with boiling for 30 min

**Table-3:** Histopathological features of liver grading criteria in OV group infection.

Histopathology	Score	Groups of experiment						

		OV	OV-PR	OV-PR5	OV-PR30	OV-PS	OV-PS5	OV-PS30
						
		N = 5	N = 5	N = 5	N = 5	N = 5	N = 5	N = 5
						
		% (N)	% (N)	% (N)	% (N)	% (N)	% (N)	% (N)
Periportal	0							
Inflammation	1				20 (1)			20 (1)
	2	80 (4)^a,b^	20 (1)	80 (4)^a^	80 (4)^a^	40 (2)	80 (4)^b^	80 (4)^b^
	3	20 (1)	80 (4)	20 (1)		60 (3)	20 (1)	
	4							
Focal	0							
Inflammation	1			40 (2)	80 (4)^a^		20 (1)	80 (4)^b^
	2	20 (1)		60 (3)^a^	20 (1)		80 (4)^b^	20 (1)
	3	80 (4)^a,b^	20 (1)			40 (2)		
	4		80 (4)			60 (3)		
Liver fibrosis	0							
	1							
	2			60 (3)	60 (3)		40 (2)	40 (2)
	3	40 (2)		40 (2)^a^	40 (2)^a^		60 (3)^b^	60 (3)^b^
	4	60 (3)^a,b^						
	5		80 (4)			60 (3)		
	6		20 (1)			40 (2)		

N=Number of hamster,

a p < 0.05 compared with OV-PR group,

b p < 0.05 compared with OV-PS group. OV=OV-infected hamsters, OV-PR=OV-infected hamsters fed PR, OV-PR5=OV-infected hamsters fed PR that had been boiled for 5 min, OV-PR30=OV -infected hamsters fed PR that had been boiled for 30 min, OV-PS=OV-infected hamsters fed PS, OV-PS5=OV-infected hamsters fed PS that had been boiled for 5 min, OV-PS30=OV-infected hamsters fed PS that had been boiled for 30 min, OV=*Opisthorchis viverrini*

### Fibrogenesis in the hamster model

**Figure-4:**
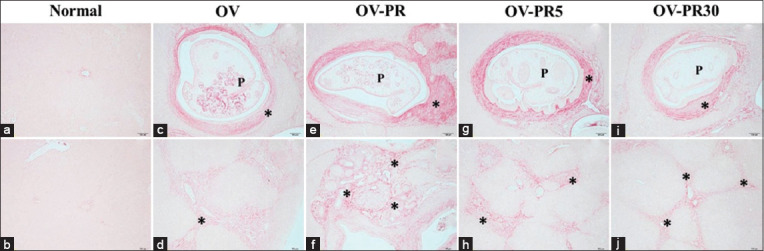
Fibrosis in the hepatobiliary system of the experimental groups. (a and b) Normal=Uninfected OV hamster, (c and d) OV=Hamster infected with *Opisthorchis viverrini*, (e and f) OV-PR=Opisthorchiasis’s hamsters feed the fermented fish, (g and h) OV-PR5=Opisthorchiasis’s hamsters feed the fermented fish with boiling for 5 min, and (i and j) OV-PR30=Opisthorchiasis’s hamsters feed the fermented fish with boiling for 30 min, P=Parasite (*O. viverrini*) in bile duct, *fibrosis.

**Figure-5:**
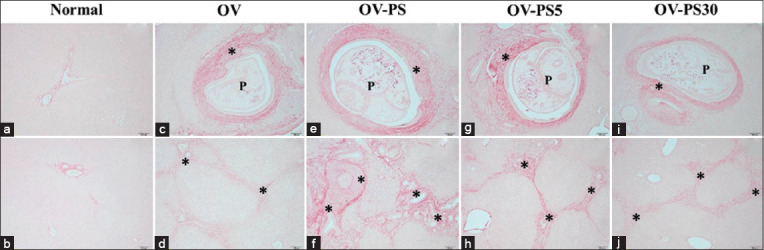
Fibrosis in the hepatobiliary system of the experimental groups. (a and b) Normal=Uninfected OV hamster, (c and d) OV=Hamster infected with *Opisthorchis viverrini*, (e and f) OV-PS=Opisthorchiasis’s hamsters feed the pickled fish, (g and h) OV-PS5=Opisthorchiasis’s hamsters feed the pickled fish with boiling for 5 min, and (i and j) OV-PS30=Opisthorchiasis’s hamsters feed the pickled fish with boiling for 30 min, P=Parasite (*O. viverrini*) in bile duct, *fibrosis.

Liver fibrosis of varying severity was present in all groups except the normal group (Figures-4a, b and 5a, b). Fibrosis was observed at the bile ducts of the portal triad in the OV group (Figures-4c, d and 5c, d). The extent of fibrosis in the OV-PR and OV-PS groups (Figures-4e, f and 5e, f) was higher than that observed in the other groups. In contrast, the groups fed the boiled fish exhibited reduced fibrosis compared with the OV-PR and OV-PS groups (Figures-4g–j and 5g–j and Table-3).

### Liver and kidney function tests

The levels of BUN, creatinine, ALP, and ALT in the N-PR and N-PS groups were significantly increased (p < 0.05) compared with the normal groups that were not fed the fermented dishes. In contrast, the BUN, creatinine, ALP, and ALT levels in the N-PR30 and N-PS30 groups were comparable with those of the normal control groups.

The levels of ALP and ALT in the OV-PR and OV-PS groups were higher than in the OV group and groups where the fermented dishes had been boiled. Notably, the levels of ALP and ALT in the OV-PR30 and OV-PS30 groups were significantly decreased (p < 0.05) when compared with the OV-PR and OV-PS groups, respectively.

The level of BUN was significantly increased in the OV-PR and OV-PS groups, as well as the OV-PR5 group. Creatinine levels were significantly increased in all fermented dish groups (Table-4).

**Table-4:** Serum level of kidney and liver function test of normal group, normal hamster treated fermented food and OV-infected groups.

Groups	Mean ± SEM			

	BUN (mg/dL)	Creatinine (mg/dL)	ALP (U/L)	ALT (U/L)
Normal	18.60 ± 0.33	0.30 ± 0.00	40.32 ± 0.59	48.74 ± 1.98
N-PR	22.73 ± 0.63*	0.40 ± 0.00*	58.33 ± 1.28*	66.00 ± 1.78*
N-PR5	23.23 ± 0.95*	0.40 ± 0.00*	50.67 ± 0.98*	64.12 ± 0.44*
N-PR30	20.40 ± 0.40	0.30 ± 0.00	43.00 ± 0.52	54.33 ± 1.02
N-PS	22.07 ± 0.97*	0.40 ± 0.00*	61.67 ± 0.88*	64.56 ± 1.02*
N-PS5	19.33 ± 0.88	0.30 ± 0.00	57.33 ± 0.81*	52.00 ± 1.08
N-PS30	18.00 ± 1.10	0.30 ± 0.00	47.01 ± 0.51	47.23 ± 1.21
OV	19.41 ± 1.34	0.30 ± 0.00	84.01 ± 1.16	104.45 ± 13.67
OV-PR	23.62 ± 1.51^#^	0.40 ± 0.13^#^	96.83 ± 3.81^#^	176.26 ± 11.65^#^
OV-PR5	22.11 ± 1.57^#^	0.40 ± 0.00^#^	91.18 ± 1.68^#^	119.42 ± 10.87
OV-PR30	19.09 ± 1.78	0.40 ± 0.00^#^	80.45 ± 2.36	104.78 ± 12.11
OV-PS	22.21 ± 1.48^#^	0.42 ± 0.07^#^	99.07 ± 2.91^#^	160.82 ± 1.08^#^
OV-PS5	20.83 ± 1.67	0.42 ± 0.09^#^	96.93 ± 3.36^#^	117.17 ± 15.09
OV-PS30	18.52 ± 1.29	0.40 ± 0.00^#^	83.86 ± 4.36	111.91 ± 17.04

Values are means ± SEM (n=5),

* p < 0.05 significant change to Normal group,

# p < 0.05 significant change to OV group. SEM=Standard deviation, N-PR=Normal hamsters feed the raw fermented fish, N-PS=Normal hamsters feed the raw pickled fish, N-PR5=Normal hamsters feed the fermented fish with boiling for 5 min, N-PS5=Normal hamsters feed the pickled fish with boiling for 5 min, N-PR30=Normal hamsters feed the fermented fish with boiling for 30 min, N-PS30=Normal hamsters feed the pickled fish with boiling for 30 min, OV=OV-infected hamsters, OV-PR=OV-infected hamsters fed PR, OV-PR5=OV-infected hamsters fed PR that had been boiled for 5 min, OV-PR30=OV-infected hamsters fed PR that had been boiled for 30 min, OV-PS=OV-infected hamsters fed PS, OV-PS5=OV-infected hamsters fed PS that had been boiled for 5 min, OV-PS30=OV-infected hamsters fed PS that had been boiled for 30 min, BUN=Blood urea nitrogen, ALT=Alanine aminotransferase, ALP=Alkaline phosphatase, OV=*Opisthorchis viverrini*

## Discussion

This is the first report of the reduction of nitrates and nitrites in fermented fish dishes following heating, as evidenced by their absence after boiling and a reduction in the aggregation of inflammatory cells surrounding hepatic bile ducts. Moreover, the levels of liver enzymes ALP and ALT were reduced.

Nitrates and nitrites are used during fermented food processing to control toxin-producing bacteria. *N*-nitroso compounds form during the preservation or fermentation processes for Chinese-style salted fish [18]. Nitrates, nitrites, and/or *N*-nitroso compounds are contaminants of fermented and pickled fish and have been found in concentrations ranging from 0 ppm to 24,354 ppm [29]. Our study found nitrate levels of <20 mg/kg and nitrite levels of <5 mg/kg in both PR and PS, which agree with the above report. Furthermore, our findings are also supported by a study on the high mortality rate of nasopharyngeal carcinoma in certain areas, which found that the level of *N*-nitroso compounds was higher (1.51 ± 0.23 mg/kg) in areas of high mortality compared with the levels (0.60 ± 0.14–0.83 ± 0.18) in areas of low mortality [30]. Recently, the presence of nitrates and nitrites in fermented food in northeast Thailand has been reported, despite controlling the levels of nitrate and nitrite salts added to the meat. Heat treatment has been employed to mitigate nitrosamine-contaminated foods. It was found that nitrosamine levels in some preserved meats, such as pork and beef, increased after cooking processes (e.g., frying, baking, and other heat treatments) [31–33]. Meanwhile, nitrosamine levels in other meats, such as horse, ram, goat, and ham pork, were decreased in previous studies [34, 35]. Heated water generates hypoxic and acidic conditions, which stimulate the physicochemical reduction reaction of NO_2_^−^ and NO_3_^−^ to gaseous nitric oxide (NO) [36], which is consequently lost to the air during the boiling process. In our study, nitrite and nitrate concentrations in PR and PS decreased when boiled. Increases in water temperature were reported to decrease water insolubility and produce hypoxic conditions [37]. Simultaneously, higher energy due to heat treatment leads to the stronger vibration of water molecules, resulting in water ionization, which produces more H^+^ and decreases pH [38].

It has been reported that the heat treatment of all raw fermented dishes resulted in high levels of inflammation and fibrosis due to NO generated by nitrite reduction [39]. Nitric oxide activates inflammation mediators, such as nuclear factor-kappa B, and it induces proinflammatory cytokines, such as tumor necrosis factor- a, which are thought to be involved in the chronic inflammatory response [40]. Chronic inflammation initiates hepatic fibrosis by promoting hepatocyte necrosis and apoptosis and then activating hepatic stellate cells and Kupffer cells, which release reactive oxygen species and NO [41].

Sriraj *et al*. [22] reported the association of OV infections from raw fermented foods (PS: fish fermented for 1 day, som wua: fermented beef, som phag: fermented vegetables, and PS: fish fermented for 6 months) with CCA development. The histopathological investigations in our study revealed aggregations of inflammatory cells in OV-infected hamsters fed raw PR and PS, as well as PR and PS that had been boiled for 30 min (OV-PR30 and OV-PS30; Figures-2 and 3). This correlated with the findings of Thongsen *et al*. [42], who reported that OV-infected hamsters showed inflammatory cell infiltration, which continues to stimulate inflammation in response to OV, but OV-infected hamsters treated with the drug praziquantel showed fewer inflammatory cells. Our findings suggest that boiling fermented dishes for 30 min (N-PR30, N-PS30) could reduce inflammatory cells or prevent CCA development.

## Conclusion

Heat treatment (i.e., boiling) reduced nitrate and nitrite concentrations and toxicity in fermented and pickled fish, as evidenced in decreased pathogenic changes in a hamster model.

## Authors’ Contributions

AA, PS, RA, AW, GNB, PB, BP, PL, PR, SB, NE, and OP: Data curation, investigation, methodology, and formal analysis. AA, PS, and TB: Conceptualization and project administration. TB and WZ: Supervision and visualization. AA, TB, PR, and GNB: Drafted and revised the manuscript. All authors have read, reviewed, and approved the final manuscript.
